# Life narratives of individuals with psychosis in ethnic minority and migrant communities in Canada and the Netherlands

**DOI:** 10.1192/j.eurpsy.2024.83

**Published:** 2024-08-27

**Authors:** I. Jansen

**Affiliations:** Vrije Universiteit Amsterdam, Amsterdam, Netherlands

## Abstract

**Background:**

Increased psychosis risk has long been reported for some migrant and ethnic minority populations, a finding has been replicated in different parts of the world, with risk seeming to persist for further generations. Several explanations such as genetic liability or selective migration, higher cannabis-use or higher exposure to neurodevelopmental risk factors were considered unlikely explanations. Rather, exposure to adversity experiences found to be a determinant of psychotic disorders, such as parental separation, social and economic disadvantage, discrimination, social exclusion and marginalization. Additionally, migrants often live in cities, where high population density, low social cohesion and social fragmentation and deprivation, combined with lack of green space and urban stress increase the psychosis risk. Although previous research work has emphasized the quantitative exploration of social-environmental determinants of psychosis, qualitative studies allow for the generation of innovative, rich and nuanced understandings about a given phenomenon, being an ideal approach in face of complex social dynamics and contexts. Concretely, the associations are established, however, the underlying mechanisms and experiences remain largely unknown.

This study aims to address several research gaps identified in research on the issues of psychosis, socio-environmental determinants of mental health, migration and ethnicity, and inequalities by exploring the life narratives and experiences of service-users with first psychosis with distinct ethnic, racial and migrant backgrounds.

**Methods:**

Participants aged between 18 and 35 years old, who have been diagnosed with a first psychosis are recruited in Montreal, Canada, and in the Netherlands. The aim is to recruit at least 20-25 individuals from each site, but recruitment is still ongoing. Qualitative interviews of about an hour are being held, and transcripts will be analyzed with Nvivo, software for qualitative data. Categories and clusters will be formed from the narratives, resulting in common themes that are important to the patients, in their understanding of the development of their psychosis, and the help they have received.

**Results:**

Preliminary data show that the patients have predominantly African or (Eastern)European background, moved around a lot, and experiences inequities. Help and care were not always available for them, not always beneficial. Participants experiences a lot of isolation and deplacement, together with socio-economic disadvantages. Common themes as to by which mechanisms these aspects play a role will further be explored.

**Discussion:**

These findings will be discussed in light of the quantitative data already existing. Implications for prevention and interventions will be discussed.

**Disclosure of Interest:**

None Declared

